# Motivational interviewing from the paediatricians’ perspective: assessments after a 2-day training for physicians caring for adolescents with chronic medical conditions (CMCs)

**DOI:** 10.1186/s12887-024-04794-z

**Published:** 2024-05-22

**Authors:** Hannah Kammering, Jennifer Antony Cruz, Anna Lena Platzbecker, Christina Reinauer, Katharina Förtsch, Lisa Krassuski, Rabea Viermann, Matthias Domhardt, Harald Baumeister, Doris Staab, Kirsten Minden, Annabel Sandra Mueller-Stierlin, Reinhard W. Holl, Petra Warschburger, Thomas Meissner

**Affiliations:** 1 University Hospital Düsseldorf, Düsseldorf, Germany; 2 Department of Clinical Psychology and Psychotherapy, Ulm University, Ulm, Germany; 3 Department of Pediatric Pneumology and Immunology, University Children’s Hospital Charité of Humboldt University, Berlin, Germany; 4 German Rheumatism Research Centre Berlin and Charité - Universitätsmedizin, Freie Universität Berlin and Humboldt - Universität zu Berlin, Berlin, Germany; 5 Department of Psychiatry II, Ulm University, Ulm, Germany; 6 Institute for Epidemiology and Medical Biometry, ZIBMT, University of Ulm, Ulm, Germany; 7 Department Psychology, Counseling Psychology, University of Potsdam, Potsdam, Germany

**Keywords:** Motivational interviewing, Adolescent, Health behaviours, Questionnaire, Counselling

## Abstract

**Background:**

Counselling adolescents with chronic medical conditions (CMCs) can be challenging regarding suitable interviewing skills and clinicians’ attitudes toward the patient. Successful communication can be a key element of treatment. Motivational Interviewing (MI) is broadly applicable in managing behavioural problems and diseases by increasing patient motivation for lifestyle changes. However, data concerning the applicability, feasibility and implementation of MI sessions in everyday practice are missing from the physicians’ point of view.

**Method:**

The present study was conducted as a mixed methods design. Twenty paediatricians were randomized to a 2-day MI course followed by MI consultations. Data were collected through a questionnaire one year after MI training. Factors for effective training and possible barriers to successful use of MI were examined.

**Results:**

Completed questionnaires were returned by 19 of 20 paediatricians. The paediatricians’ experiences with MI demonstrate that MI is regarded as a valuable tool when working with adolescents with CMCs. 95% of all respondents reported that they found MI education necessary for their clinical work and were using it also outside the COACH-MI study context. 73.7% percent saw potential to strengthen the connection to their patients by using MI. The doctors were already using more MI conversation techniques after a 2-day MI course. Obstacles were seen in the short training, the lack of time and missing undisturbed environment (interruptions by telephone, staff, etc.) during clinical flow.

**Conclusions:**

MI techniques are not yet a regular part of medical training. However, a 2-day MI course was rated effective and provided a lasting impact by physicians caring for children and adolescents with chronic medical conditions (CMCs), although booster sessions should be offered regularly.

**Trial registration:**

The study was registered in the German Clinical Trials Register (DRKS00014043) on 26/04/2018.

## Introduction

Children and adolescents with chronic medical conditions (CMCs) have an elevated risk of developing psychological comorbidities, such as anxiety and depression [1–6]. In addition to concerns about the diagnosis and prognosis, regular long-term treatments affect the daily lives of CMCs. Among social disturbances, stigmatisation and rejection by peers are a major challenge that can have a negative impact on self-confidence and self-esteem [2].

The effectiveness of integrated mental health care in paediatric settings has received increased attention [7]. More specifically, validated diagnostic instruments and brief psychological interventions, such as Motivational Interviewing (MI) for behavioural change, were shown to improve primary clinical outcomes and mental health symptoms [8, 9]. In this context, good co-operation between paediatricians and patients and a corresponding communicative competence of the paediatricians would be desirable. MI is a client-centered conversation technique and a directive approach to explore ambivalence and develop intrinsic motivation purposefully. Building on a patient empowerment perspective, MI has emerged as an effective counselling technique to detect comorbid mental health problems and support health-related lifestyle changes [10–14]. In MI conversations, various techniques are used, such as open-ended questions, active listening, providing confirmation, summarizing, affirming, and reflecting on behaviour [7, 9]. The aim is eliciting”Change talk” and “Confidence talk” to bring about behaviour changes [10]. Change talk includes any statement by the doctor that favours a movement towards a specific change goal, while confidence talk expresses in particular the ability to change. MI was initially used to treat addictive behaviour and has been used for several other behavioural changes (e.g. health behaviour and health service use) in the meantime [11]. Furthermore, it was shown that MI improves the utilization of psychiatric care services by young patients [12, 15–20]. Published data suggests implementing MI techniques into clinical practice to be feasible, as even 15-minute counselling applying MI techniques can be effective [14].

Physicians can acquire MI techniques in professional training sessions [21, 22]. A review of ten studies by Söderlund et al. [23] found an average initial training duration of nine hours for general health care practitioners in learning MI techniques. Significant improvement in the long-term quality of MI was achieved through regular follow-up sessions. Most training courses are offered in the format of one- to three-day workshops, emphasizing the importance of continuous follow-up training, e.g., in the form of supervision [21, 22].

To date, few studies have addressed and systematically analyzed experiences with MI from the physicians’ perspective. This study aimed to fill this knowledge gap and to provide recommendations for the integration of MI into the clinical routine in the care of adolescents. Therefore, we investigated.

- paediatricians’ experiences with a 2-day basic MI education,
- paediatricians’ experiences using MI as part of the single-center cluster-randomized controlled COACH-MI trial [24] to improve uptake of mental health care for adolescents with CMCs and comorbid symptoms of anxiety and depression,
- paediatricians’ experiences integrating MI into the daily clinical practice of paediatricians caring for chronically ill adolescents at a University children’s hospital outpatient clinic.

## Methods

The study was conducted within the multicenter project of the COACH consortium (Chronic Conditions in Adolescents: Implementation and Evaluation of Patient-Centered Collaborative Health Care), aiming to improve awareness and access to mental health care for adolescents with CMCs. In this cluster-randomized trial with 164 adolescents with CMCs and comorbid anxiety or depression, training physicians in MI improved uptake rates of psychological counselling among adolescents, however results did not reach statistical significance [24]. Our study was conducted following the main study [24] from May to August 2021.

### Aims

Our aim was to explore clinicians’ experiences of MI training and subsequent use of MI in the routine care of adolescents with CMCs. Therefore, we wanted to find out if and how MI can be integrated into clinical practice and how training in MI should be designed.

### Design

A mixed methods study approach with quantitative and qualitative data gathered with based on a pseudonymized questionnaire was employed to explore the opinions, experiences, and needs of paediatricians using MI in everyday practice.

### Participants and setting

The COACH-MI trial was conducted at the outpatient clinics of the University Children’s Hospital Düsseldorf, Germany (Endocrinology and Diabetes, Pulmonology, Cardiology, Gastroenterology, Neurology, Immunology and Rheumatology, Metabolism), as described previously [24]. After completion of the main study [24], our study was conducted between May and August 2021. Out of 25 physicians, 20 participated in the project. Five physicians left the outpatient department or the hospital before completing the first MI session. As part of the study, the doctors attended a 2-day in-person MI training course, conducted by a Motivational Interviewing Network of Trainers (MINT) certified trainer [25] and booster sessions one year after study initiation. None of the paediatricians had previous specialized training in psychiatry, psychotherapy, or MI prior to study start. The aim was to collect data from the doctors’ perspective on their experiences with the MI technique; the response rate was 95% (19/20).

### Data collection

A self-report questionnaire gathered data on the following themes: MI skills/proficiency, actual MI use in everyday practice, opinions on MI, and need for training and framework conditions in clinical routine. No validated questionnaire was available for evaluating experiences with MI and the physicians’ perception of the method, the technique, and the application of MI in clinical practice. Therefore, the questionnaire was developed by our study team. One author, who has a strong background in educational theory and questionnaire design, and two other authors - a total of 2 paediatricians and a psychologist - developed the questionnaire in German language and included a total of 16 questions on the above-mentioned themes. As there was no validated questionnaire in this topic, we developed questions which relate to factors that could be important based on our experience and informal discussions with doctors.

The three-page questionnaire collected demographic and practice information, such as age, gender, qualification, and work experience in order, to characterize the sample of paediatricians. We used different question types: closed questions (yes/no), open questions, and rating scales (linear Likert scale). The questionnaire asked respondents to rate on a six-point Likert scale the extent to which of nine MI conversation techniques were used before and after MI training. We chose a bipolar Likert scale to reflect the agreement or disagreement on a 6-point scale to avoid a neutral middle option. The questionnaire is reliable. The Cronbach’s alpha value for the nine items measuring the dialogue techniques used before and after the MI training is 0.860. Open-ended questions asked for suggestions to make MI better using in everyday clinical practice and for general comments. Questionnaires were completed anonymously to preserve participant privacy. The answers to open-ended questions were analyzed and assigned to labels by the first author of this paper.

## Results

### Study conduct

Consent and complete questionnaires were provided by *n* = 19 of 20 paediatricians (response rate of 95%), while one physician did not “consent” to participate in the study. Of these, *n* = 12 (63.2%) participants were female, *n* = 7 (36.8%) male, *n* = 3 (15.8%) participants were in residency training, *n* = 9 (47.7%) were specialists, and *n* = 7 (36.8%) were senior physicians. The average work experience was 12.2 years.

### Personal experiences

**Fig. 1:**
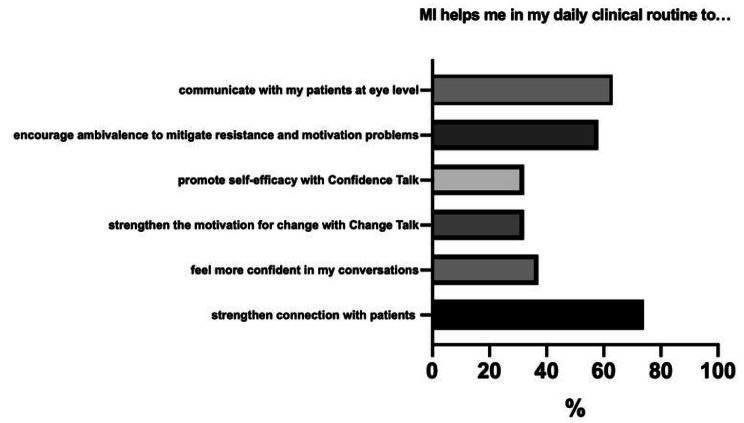
Physicians’ view on benefits of MI application in clinical routine

**Fig. 2:**
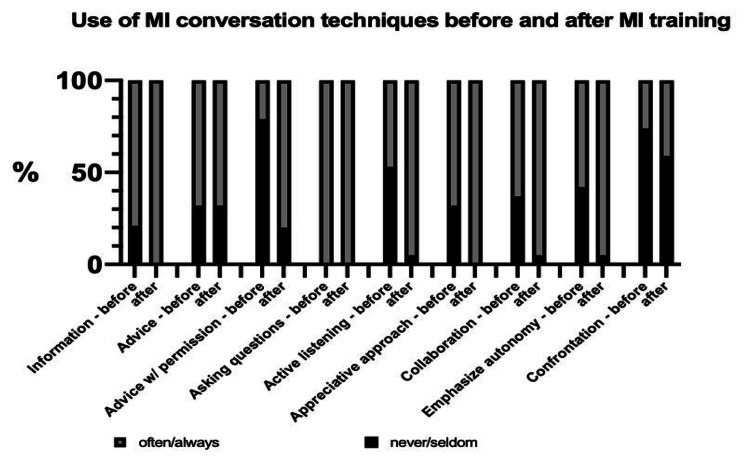
Use of MI techniques before and after MI training

The vast majority of all respondents (94.7%) reported that they found MI helpful for clinical conversations. They stated it was important for their clinical work (Likert scale from 1 = *not important* to 6 = *very important*; *M* = 4.7, SD 1.2) and used it outside the COACH-MI study context (Likert scale from 1 = *never* to 6 = *always*; *M* = 4.1, SD 1.0). *n* = 7 (36.8%) physicians stated they felt more secure during patient conversations using MI techniques. *n* = 14 physicians (73.7%) thought MI strengthened the physician-patient-alliance. About two-thirds (*n* = 12; 63%) of the respondents perceived that conversations are conducted “on equal terms” with the adolescents by using MI techniques, and *n* = 11 (58%) physicians promoted confidence talk. About one-third (*n* = 6; 32%) promoted change talk and resolved ambivalences in their patients (Fig. 1). Concerning MI training, more MI techniques were used after training (Likert scale from 1 *never* to 6 *always;* before *M* = 3.7, SD 1.3 vs. after *M* = 4.5, SD 1.1). Primarily the following methods were increasingly applied: advising with permission (*M =* 2.5, SD 1.5 vs. *M =* 4.3, SD 1.1), reflective listening (*M =* 3.4, SD 1.2 vs. *M =* 4.8, SD 0.9), an appreciative approach (*M =* 3.8, SD 1.3 vs. *M =* 5, SD 0.8), and emphasizing autonomy (*M =* 3.7, SD 1.2 vs. *M =* 4.6, SD 0.8) (Fig. 2). The following groups of patients were perceived to benefit most from MI: adolescents (47.4%), patients with CMCs (47.9%), and patients with noncompliance (26.3%). Here, respondents indicated that MI is beneficial for crisis conversation (52.6%), as well as compliance issues (31.6%) and first consultations (26.3%). It was perceived as less helpful in informed consent discussions (15.8%) and follow-up discussions (10.5%).

### External and internal framework conditions

**Fig. 3:**
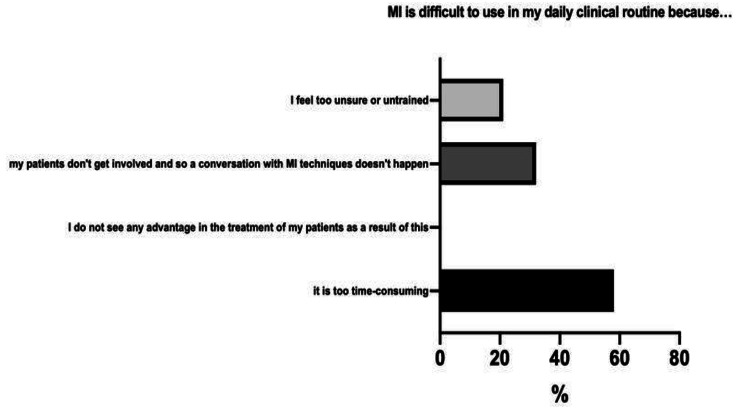
Physicians’ impediments to use MI in clinical routine

About one-third (*n =* 6; 31.6%) stated that insufficient framework conditions hampered MI conversations. Due to lack of time, only half of the paediatricians (*n =* 9; 47.4%) offered second appointments to discuss critical topics further, although *n* = 17 (89.5%) stated that more appointments (> 1 appointment) would have been needed for sufficient MI application. To overcome the aforementioned barriers in clinical practice, respondents indicated the most important factor to be a distraction-free environment, specifically a calm, quiet room, no disturbance from other staff and calls (57.9%; Fig. 3), as well as more scheduled time for patient-conversations (36.8%). On average, physicians reported that their MI conversations lasted about 25 min. In addition, *n =* 4 (21.1%) of the respondents thought that important general conditions were establishing a safe environment for the patient to speak freely. Only n *=* 2 (10.5%) physicians stated that they had too little practical experience and did not feel sufficiently trained. Therefore, *n* = 4 (21.1%) physicians felt insecure about conducting MI consultations (Fig. 3).

### Training

**Fig. 4:**
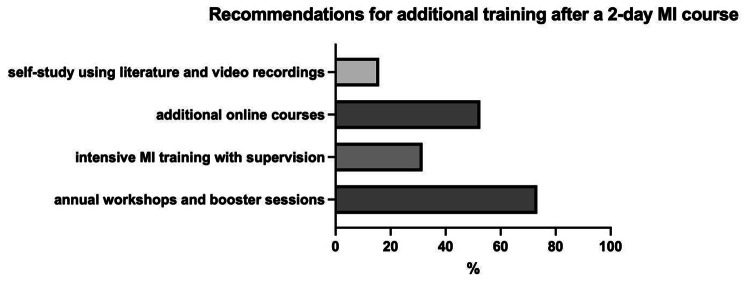
Recommendations for additional training after a 2-day course

All doctors have completed a 2-day course learning MI. More than half of the doctors (57.9%) felt that the training was sufficient to train the basics, however, they wanted additional interventions, e.g. in the context of booster sessions. Most of the respondents (73.7%) recommended annual workshops and booster sessions. *N =* 6 (31.6%) of the respondents wished for more intensive MI training with supervision, with about half (*n =* 10; 52.6%) suggesting training via online courses. Only *n =* 3 (15.8%) preferred self-study using literature and video recordings. These results are presented in Fig. 4. The respondents stated that MI training is important for residency (Likert scale from 1 = *not important* to 6 = *very important*; *M* = 4.7, SD 1.2), and *n =* 18 (94.7%) respondents stated that MI training should be integrated into residency training. Additionally, *n =* 12 (63.2%) wished for earlier conversation training during medical school, and *n =* 10 (52.6%) paediatricians recommended further training after residency.

## Discussion

There are several reasons for physicians to improve their conversational skills and attitude in communicating with patients. This might be especially true when dealing with adolescents with chronic medical conditions e.g., type 1 diabetes, rheumatic diseases, neurological disorders, gastrointestinal diseases, or congenital heart diseases. In our main study [24], we were able to show that the use of MI in patients with CMCs leads to longer patient-physician conversations and lower anxiety scores at one year. We evaluated paediatricians’ experiences with MI after a 2-day workshop and opportunities and challenges in terms of MI integration into everyday clinical practice.

Paediatricians working in outpatient clinics generally considered MI helpful. In line with the results of Rubak et al. [14] and Reinauer et al. [24], MI was perceived to have a positive impact on physician-patient interactions, compared to traditional counselling. In line with previously published literature, participating physicians felt more confident by using MI techniques [26]. Integrating MI into clinical practice comes with several challenges. Our results support previously published findings that MI requires a time frame that is not always available in routine patient care [21, 27–30]. In our study, MI conversations to discuss a conspicuous mental screening result lasted an estimated 30,3 min [24]. In the study here, the mean conversation time was estimated to be 25 min. The discrepancy between these two times is due to the fact that the questionnaires were completed after one year. The investigators stated that they needed more time or more appointments to talk to their patients, but that this was often not feasible in the daily clinical routine due to timetabled structures. In a study by Kirschner et al. [31] lack of time was also mentioned as a major obstacle. MI training was associated with longer patients-physician conversations. MI conversations were significantly longer than TAU (30.3 [16.7] vs. 16.8 [12.5] min; *p* < 0.001) [24]. Additionally, half of the paediatricians scheduled second appointments with patients to sufficiently apply MI techniques. Other studies [14, 32] have shown that even short interventions of about 15 min can affect behavioural changes in patients. The likelihood of behavioural change increases with the number of conversations scheduled [14, 32]. Some general aggravating conditions were criticized in our study. The MI conversations occurred in consulting rooms with disturbances, such as entering staff or ringing telephones. Therefore, an uninterrupted atmosphere was perceived as an essential factor for implementing MI.

After two days of MI training, the use of MI was still found to be challenging by part of the trained physicians, and regular training was suggested to avoid falling back into old patterns of behaviour. Some physicians reported feeling insecure in their MI proficiency, regardless of whether they had attended a booster session or not. More than half of the doctors (57.9%) felt that the training was not sufficient and would have liked further interventions to practice MI, such as booster sessions. Past research has demonstrated the importance of close integration of training and practice [21]. Keeley et al. [33] conducted a study offering baseline training plus two refresher training courses of 4 h each, along with feedback on audiotaped patient encounters. This study elaborated the importance of follow-up training as basic courses alone may not be sufficient to reach MI proficiency. Miller et al. [34] investigated the effect of feedback and coaching after a 2-day basic course and the impact of self-study through training videos after a 2-day basic course: No improvement in the performance of MI was achieved through self-study. However, with regular feedback and coaching, MI skills could be consolidated and maintained. A meta-analysis by de Roten et al. [35] supported the improvement of MI skills by adding feedback in the context of supervision or coaching. Lindhardt et al. [22], Miller et al. [34], and Brobeck et al. [27] also state the importance of supervision and follow-up sessions. Surprisingly, only *n =* 6 (31.6%) of the study physicians indicated that supervision was helpful. Most physicians (*n =* 10; 52.6%) considered 2-day basic training and booster sessions sufficient, and would have additionally considered online courses useful. The participants probably included the feasibility of specific MI training techniques in everyday practice in their judgment. Due to time constraints, they might find supervision to be too time-consuming. Nevertheless, we were able to demonstrate that a 2-day course led to changes in the applied conversation techniques, which is in line with published data [13, 32]. The patients seem to benefit more from the intervention with increasing MI experience [36].

Notably, nearly all of the physicians participating in our study felt that it was important for MI training to be integrated into residency training, and a majority thought it would be necessary to start training during medical school as well. Most studies concentrate on medical staff such as doctors, nurses, and midwives, as conducted by Madson et al. [37]. Poirier et al. [38] demonstrated that teaching motivational interviewing techniques to first-year medical students can enhance student knowledge and confidence in patient counselling regarding health behaviour changes. Therefore, it seems reasonable to implement MI training early in medical staff education.

### Limitations

When interpreting the results, some limitations must be taken into account. On the one hand, a limited number of paediatricians were recruited in our single-center study. On the other hand, no validated questionnaire was available for evaluating paediatricians’ experiences with a two-day MI workshop. Thus, the questionnaire was designed to address our research questions. The different questions (open questions, closed questions…) as well as the wording of the questions can have a potential influence on the answers of the doctors surveyed. As our questionnaire is not scientifically validated, but was developed by ourselves, the occurrence of various confounding factors cannot be ruled out and should be taken into account when interpreting the results. These confounding factors include the different question types described above, but also the different possible interpretations of the question and/or the possible answers. Furthermore, this questionnaire is not a generally valid questionnaire for surveying MI technique for various professional sectors, but is specifically aimed at doctors. The application of MI in the study was limited to counselling adolescents with CMCs and a positive screening for anxiety and depression symptoms. The current questionnaire was conducted one year after the COACH-MI study was completed, and this temporal distance might have influenced the physicians’ responses and might incur substantial recall bias. Further, querying paediatricians about their practices pre- and post-MI training, knowing the MI-training is the studied intervention, is prone to social desirability bias.

### Future directions

Comprehensive integration of MI into the clinical routine of physicians treating chronically ill adolescents is challenging. This is traced back to the lack of time and space resources in the clinical routine at a University outpatient clinic for the practice of MI and the lack of continued acquisition of sufficient training skills. Future research is needed to determine whether supervised sessions are accepted to improve physician education, if a corresponding time frame is made possible. Future research should focus not only on MI training but also on the implementation process in clinical settings, especially when time resources are limited.

## Conclusion

According to physicians who care for chronically ill adolescents, even a 2-day MI training course can sustainably improve communication behaviour with this patient group. The need to integrate basic knowledge (of MI) into the training of physicians at an early stage has become obvious, as well as to offer more advanced training opportunities and time resources to experienced physicians. Overall, it would make sense to implement MI as a fixed treatment component in the daily routine care of healthcare systems, although the lack of a time component and an undisturbed environment are seen as the main obstacles to implementation.

## Data Availability

The data that support the findings of this study are available on request from the corresponding author. The data are not publicly available due to privacy or ethical restrictions. Individual de-identified, anonymized data are available from the authors upon reasonable request (https://www.coach.klips-ulm.de/).

## Abbreviations

CMCs: Chronical medical conditions
COACH: Chronic Conditions in Adolescents: Implementation and Evaluation of Patient-Centered Collaborative Healthcare
DRKS: German Clinical Trials Register
MI: Motivational interviewing
MINT: Motivational Interviewing Network of Trainers
TAU: Treatment As Usual
